# Genetic effect of metformin use on risk of cancers: evidence from Mendelian randomization analysis

**DOI:** 10.1186/s13098-023-01218-3

**Published:** 2023-12-06

**Authors:** Yao Chen, Bingjun Bai, Shuchang Ye, Xing Gao, Xinnan Zheng, Kangkang Ying, Hongming Pan, Binbin Xie

**Affiliations:** 1 Department of Medical Oncology, Sir Run Run Shaw Hospital, School of Medicine, Zhejiang University, 3# East Qingchun Road, Hangzhou, 310016 Zhejiang People’s Republic of China; 2 Department of Colorectal Surgery, Sir Run Run Shaw Hospital, School of Medicine, Zhejiang University, Hangzhou, 310016 People’s Republic of China; 3 Department of Cardiology, The Second Affiliated Hospital, School of Medicine, Zhejiang University, Hangzhou, 310016 People’s Republic of China; 4 Department of Oncology, The Second Affiliated Hospital, Soochow University, Suzhou, 215004 People’s Republic of China; 5 Department of Radiation Oncology, Women’s Hospital, School of Medicine, Zhejiang University, Hangzhou, 310006 People’s Republic of China

**Keywords:** Metformin, Cancers, Genetic association, Mendelian randomization, Testosterone

## Abstract

**Background:**

Increasing number of studies reported the positive effect of metformin on the prevention and treatment of cancers. However, the genetic causal effect of metformin utilization on the risk of common cancers was not completely demonstrated.

**Methods:**

Two-sample Mendelian Randomization (two-sample MR) analysis was conducted to uncover the genetically predicted causal association between metformin use and 26 kinds of cancers. Besides, two-step Mendelian Randomization (two-step MR) assessment was applied to clarify the mediators which mediated the causal effect of metformin on certain cancer. We utilized five robust analytical methods, in which the inverse variance weighting (IVW) method served as the major one. Sensitivity, pleiotropy, and heterogeneity were assessed. The genetic statistics of exposure, outcomes, and mediators were downloaded from publicly available datasets, including the Open Genome-Wide Association Study (GWAS), FinnGen consortium (FinnGen), and UK Biobank (UKB).

**Results:**

Among 26 kinds of common cancers, HER-positive breast cancer was presented with a significant causal relationship with metformin use [Beta: − 4.0982; OR: 0.0166 (95% CI: 0.0008, 0.3376); *P *value: 0.0077], which indicated metformin could prevent people from HER-positive breast cancer. Other cancers only showed modest associations with metformin use. Potential mediators were included in two-step MR, among which total testosterone levels (mediating effect: 24.52%) displayed significant mediating roles. Leave-one-out, MR-Egger, and MR-PRESSO analyses produced consistent outcomes.

**Conclusion:**

Metformin use exhibited a genetically protective effect on HER-positive breast cancer, which was partially mediated by total testosterone levels.

**Supplementary Information:**

The online version contains supplementary material available at 10.1186/s13098-023-01218-3.

## Introduction

The growing frequency and high mortality of malignant tumors imposed a significant burden on people all over the world [1]. Over the past few decades, healthcare professionals have tirelessly sought effective and safe strategies for cancer prevention, albeit with limited success. Metformin, a widely prescribed medication for managing type 2 diabetes, has garnered increasing interest for its potential anti-tumorigenic properties [2]. An increasing number of clinical studies attempted to reveal the efficacy of metformin on different types of cancer, but the controversial conclusions left the issue unsolved [3–5]. Biases induced by confounders which were hard to avoid in observational studies might be responsible for this.

Scientists were also interested in the biological pathways of metformin in cancer treatment. Apart from its well-documented benefits in improving glucose metabolism, recent years have witnessed extensive investigations into metformin's molecular mechanisms against various malignancies. These mechanisms include the reduction of leukocyte–endothelium interactions, modulation of oxidative stress, and the regulation of AMP-activated protein kinase (AMPK) [6, 7]. However, researches related to the genetic effect of metformin use on the risk of cancers were not complete yet.

Contrary to conventional observational research, Mendelian Randomization analysis (MR) provided a cost- and time-saving approach with high efficiency to investigate the genetically predicted causal relationships [8]. As Dr. Tobin stated, MR is also known as 'Mendelian deconfounding' since it attempts to present estimates of causal effects that are free of biases caused by confounding [9]. Robust-associated genetic variants were selected to explore the genetic association between exposures and outcomes. Genetic variations in the MR method are equal to lifetime changes caused by exposure and reflect the long-term implications of the alteration on certain illnesses [10]. In our study, two-sample MR was used to unveil the causal relationship between metformin use and 26 common types of cancer. Additionally, we supplemented our analysis with a two-step MR to identify potential mediators and assess their contribution to the genetic causal effect. Ultimately, we conducted a comprehensive review of previous clinical studies to enhance our understanding of the association between metformin use and cancer.

## Materials and methods

### Study design

**Fig. 1:**
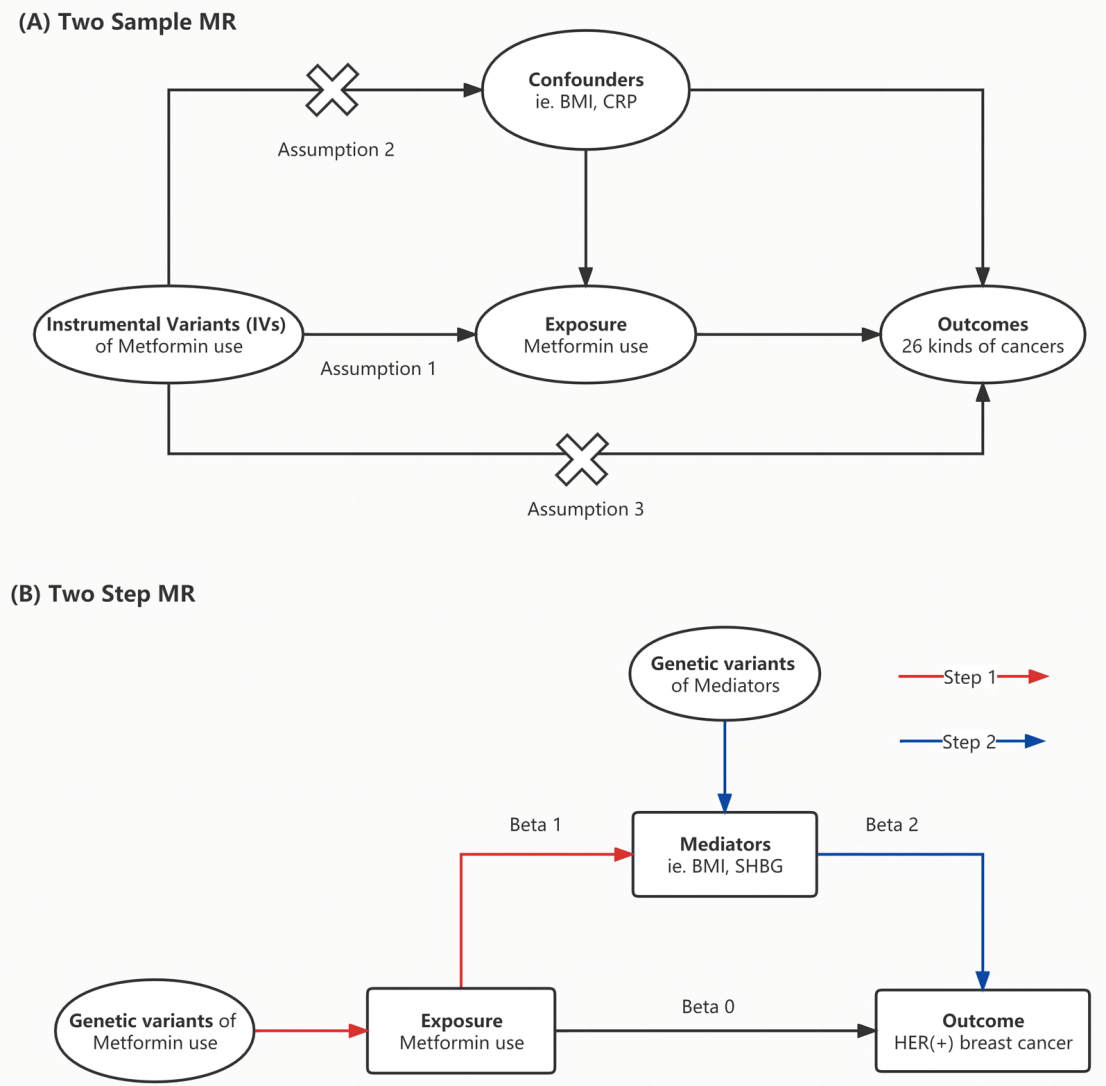
Overview of the study design. **A** We firstly applied two-sample MR analyses to figure out the genetic effect of metformin use on 26 prevalent cancers with five robust methods. **B** Two-step MR analysis and MVMR were further conducted to figure out the potential mediator who mediate the protective genetic effect of metformin on HER-positive breast cancer

The overview of the study design is demonstrated in Fig. 1. As shown in Fig. 1a, the MR analysis requires three basic assumptions to be met: (1) instrumental variables (IVs) are strongly correlated to exposure; (2) IVs are independent of any potential confounders; and (3) IVs only affect the outcome through exposure. Two distinct genetic datasets should be integrated into a single MR study, which is the fundamental prerequisite of two-sample MR.

To determine the mediating factors in the genetic causal relationship, two-step MR was performed as illustrated in Fig. 1b. In the first step, single-nucleotide polymorphisms (SNPs) to genetically predict metformin use were incorporated to evaluate the causal relationship of metformin use on 22 potential mediators (e.g. BMI, CRP, and testosterone levels) in the univariable MR method. And SNPs robustly related to mediators were used to calculate the causal association of mediators and cancer outcome(s) [11]. It should be noted that the genetic information utilized in this study is freely accessible to researchers around the world and is therefore not subject to additional ethical review or informed consent.

### Selection of instrumental variants (IVs) of metformin use

Genetic variants of metformin use in European ancestry were obtained from the UK Biobank dataset (8392 cases/328,767 controls). The following inclusion criteria guided our selection of the IVs: (1) SNPs should have a genome-wide significance level (*P* < 5×10^–8^), which strongly indicates genetic association with exposure. (2) Genetic variants with linkage disequilibrium (LD) (r^2^ > 0.001) were excluded. The LD between SNPs was assessed to clump the independence of SNPs; (3) The F-statistics (beta2/se2) > 10. SNPs with F-statistics less than 10 may have inferior statistical power. Additional file 1: Table S1 summarizes the IVs of metformin use involved in this work.

### Selection of cancer outcomes

The genetic information associated with the following types of malignant tumors was obtained from the FinnGen consortium: colorectal cancer (3022 cases/215,770 controls), stomach cancer (633 cases/218159 controls), pancreas cancer (605 cases/218187 controls), oral pharynx cancer (126 cases/218666 controls), oesophagus cancer (212 cases/218560 controls), bone and articular cartilage cancer (119 cases/218673 controls), kidney cancer (971 cases/217,821 controls), melanoma (98 cases/218694 controls), non-melanoma skin cancer (10,382 cases/208410 controls), thyroid gland cancer (989 cases/217803 controls), overall breast cancer (8401 cases/115178 controls), HER-negative breast cancer (3092 cases/99267 controls), HER-positive breast cancer (4263 cases/99267 controls), lung cancer (1681 cases, 217,111 controls), non-small cell lung cancer (NSCLC) (1627 cases/217165 controls) and small cell lung cancer (SCLC) (179 cases/218613 controls). The genetic information of other cancers was gotten from UKB: colon cancer (2226 cases/358968 controls), rectum cancer (1085 cases/461925 controls), liver cancer (168 cases/372016 controls), small intestine cancer (156 cases/337003 controls), bladder cancer (1554 cases/359640 controls), overall skin cancer (1436 cases/461497 controls). Only the European population was incorporated into this study, and no sample overlap in this MR study.

### Statistical analyses

#### Two-sample Mendelian randomization

Two-sample MR studies were conducted using TwoSampleMR package (version 0.5.6) and R software (version 4.2.1) [12]. A total of five different approaches were used. The inverse variance weighting (IVW) method, which evaluates the causal influence of genetically predicted exposures on outcomes by weighted regression of SNP-specific Wald ratios, acted as the major approach. To examine the consistency and heterogeneity of our findings, four additional assessment techniques—weighted median, MR Egger, simple model, and weighted model—were performed [13–15]. When the variable in MR has an impact on illness independent of its impact on exposure, this is known as horizontal pleiotropy. To avoid the biases of horizontal pleiotropy, MR-PRESSO method was performed to identify the outliers with MRPRESSO package (version 1.0) [16]. Pleiotropy was tested by leave-one-out analysis and MR-Egger intercept method [17, 18]. Heterogeneity was evaluated by Cochran's Q-statistic, and any MR results with heterogeneity were excluded.

#### Median analysis

The genetic information of potential mediators was downloaded from publicly accessible GWAS consortia, and relevant GWAS identifiers or available references were listed in Table 2. Two-step MR analysis was applied to figure out if the potential mediator attributed any mediating effect between exposure and outcome [11]. Of note, the mediator has to meet the premise of a continuous variable [19]. In the first step, genetic variants of exposure (metformin use) were obtained to determine the causal effect of exposure on potential mediators. After that, genetic variants of mediators were also acquired to assess the causal role of mediators on outcomes (cancers) in the second step. Beta 1 and beta 2 were calculated in step one and step two respectively (Fig. 1b). Potential mediator which presented supporting evidence in two-step MR would be included in the median analysis. Multivariable MR (MVMR) analysis was performed on metformin use-TT level-HER(+) breast cancer. The mediation effect was obtained by multiplying beta1 by beta 2.

### Comparison with clinical studies

To further confirm our findings, we reviewed the prevention and treatment effects of metformin on breast cancer in previous clinical studies. Phrase II, Phrase III randomized clinical trials (RCT), prospective studies and retrospective studies that published on Pubmed, Medline and Embase were included.

## Results

### Selected genetic instrumental variants (IVs)

We meticulously followed the aforementioned criteria when selecting the IVs. As a consequence, 26 independent SNPs were selected out of the total amount of 10,894,596 SNPs, acting as the IVs of metformin use. Detailed information could be found in Additional file 1: Table S1. F-statistics, which were also presented in the supplementary document, showed no evidence of weak instrumental bias.

### Assessment of the genetic causal effect of metformin on cancers

#### Two-sample MR results

The brief results of two-sample MR analyses of metformin use on 26 prevalent cancers were listed in Table 1. IVW results presented the genetically predicted protective effect of metformin use on HER-positive breast cancer (Beta: − 4.0982; OR: 0.0166 (95% CI: 0.0008, 0.3376); *P *value: 0.0077). The scatter plots and funnel plots were illustrated in Additional file 1: Fig. S1. The leave-one-out analysis showed no pleiotropy in the MR result (Additional file 1: Fig. S2). And no significant genetic relationship existed between metformin use and other types of cancers.

**Table 1:** Two-sample Mendelian randomization results for genetic effect of metformin use on 26 common cancers

Outcomes	IVs amount	Methods	Beta	Odds ratio	OR_lo95%	OR_up95%	*P *value	Q-statistics	*P*_h_-value
*Gastrointestinal tract cancers*									
Colorectal	24	MR Egger	− 6.21E+00	2.01E−03	1.43E−07	2.83E+01	2.16E−01	2.88E+01	1.49E−01
		Weighted median	− 2.73E+00	6.53E−02	2.41E−04	1.77E+01	3.40E−01		
		IVW	− 2.70E+00	6.71E−02	1.27E−03	3.55E+00	1.82E−01	2.97E+01	1.59E−01
Colon	25	MR Egger	6.92E−03	1.01E+00	9.44E−01	1.07E+00	8.36E−01	2.74E+01	2.39E−01
		Weighted median	1.18E−02	1.01E+00	9.72E−01	1.05E+00	5.64E−01		
		IVW	1.20E−02	1.01E+00	9.85E−01	1.04E+00	3.86E−01	2.74E+01	2.84E−01
Rectum	9	MR Egger	− 2.11E−01	8.09E−01	6.33E−01	1.04E+00	1.36E−01	1.04E+01	1.68E−01
		Weighted median	− 1.43E−02	9.86E−01	9.50E−01	1.02E+00	4.49E−01		
		IVW	− 1.14E−02	9.89E−01	9.56E−01	1.02E+00	5.01E−01	1.42E+01	7.68E−02
Liver	19	MR Egger	6.22E−03	1.01E+00	9.86E−01	1.03E+00	5.59E−01	2.55E+01	8.40E−02
		Weighted median	− 2.30E−03	9.98E−01	9.87E−01	1.01E+00	6.75E−01		
		IVW	− 3.19E−03	9.97E−01	9.88E−01	1.01E+00	4.70E−01	2.70E+01	7.93E−02
Stomach	24	MR Egger	− 1.21E+01	5.50E−06	9.34E−14	3.24E+02	1.98E−01	1.31E+01	9.31E−01
		Weighted median	− 4.97E+00	6.97E−03	2.86E−07	1.70E+02	3.35E−01		
		IVW	− 2.76E+00	6.34E−02	3.68E−05	1.09E+02	4.68E−01	1.43E+01	9.17E−01
Pancreas	24	MR Egger	3.36E+00	2.88E+01	1.23E−08	6.75E+10	7.63E−01	3.03E+01	1.12E−01
		Weighted median	5.58E+00	2.64E+02	1.25E−03	5.59E+07	3.73E−01		
		IVW	3.47E+00	3.21E+01	4.92E−03	2.10E+05	4.39E−01	3.03E+01	1.42E−01
Small intestine	25	MR Egger	3.20E−04	1.00E+00	9.84E−01	1.02E+00	9.69E−01	1.96E+01	6.68E−01
		Weighted median	3.15E−03	1.00E+00	9.93E−01	1.01E+00	5.58E−01		
		IVW	1.72E−03	1.00E+00	9.95E−01	1.01E+00	6.19E−01	1.96E+01	7.20E−01
Larynx	24	MR Egger	3.50E+00	3.31E+01	1.13E−13	9.68E+15	8.39E−01	2.12E+01	5.10E−01
		Weighted median	8.72E+00	6.11E+03	2.23E−06	1.68E+13	4.32E−01		
		IVW	4.62E+00	1.02E+02	9.25E−05	1.11E+08	5.15E−01	2.12E+01	5.70E−01
Oral pharynx	24	MR Egger	− 1.72E+01	3.26E−08	1.66E−25	6.38E+09	4.05E−01	2.26E+01	4.24E−01
		Weighted median	− 1.16E+01	9.10E−06	1.35E−16	6.11E+05	3.61E−01		
		IVW	− 1.25E+01	3.86E−06	2.82E−13	5.29E+01	1.37E−01	2.27E+01	4.79E−01
Oesophagus	24	MR Egger	2.15E+01	2.14E+09	4.16E−04	1.10E+22	1.64E−01	2.06E+01	5.46E−01
		Weighted median	8.05E+00	3.14E+03	2.61E−05	3.79E+11	3.96E−01		
		IVW	1.15E+01	9.51E+04	4.68E−01	1.93E+10	6.60E−02	2.11E+01	5.73E−01
*Breast cancers*									
Breast cancer	24	MR Egger	6.05E−01	1.83E+00	2.06E−03	1.63E+03	8.63E−01	3.46E+01	4.22E−02
		Weighted median	− 1.10E+00	3.32E−01	1.28E−02	8.65E+00	5.08E−01		
		IVW	− 2.26E+00	1.05E−01	6.17E−03	1.77E+00	1.18E−01	3.59E+01	4.18E−02
HER(−)	24	MR Egger	3.37E+00	2.91E+01	3.84E−04	2.21E+06	5.62E−01	3.89E+01	1.45E−02
		Weighted median	− 1.33E+00	2.65E−01	1.12E−03	6.27E+01	6.34E−01		
		IVW	− 2.00E+00	1.36E−01	1.23E−03	1.50E+01	4.06E−01	4.08E+01	1.26E−02
HER(+)	24	MR Egger	− 1.16E+00	3.14E−01	2.35E−04	4.19E+02	7.55E−01	1.63E+01	7.99E−01
		Weighted median	− 2.42E+00	8.88E−02	1.11E−03	7.09E+00	2.79E−01		
		IVW	− 4.10E+00	1.66E−02	8.16E−04	3.38E−01	7.66E−03*	1.71E+01	8.04E−01
ER(+)	24	MR Egger	3.59E+00	3.61E+01	1.33E−01	9.78E+03	2.23E−01	1.15E+02	1.68E−14
		Weighted median	− 2.00E−01	8.19E−01	1.18E−01	5.68E+00	8.40E−01		
		IVW	− 1.19E+00	3.04E−01	2.88E−02	3.22E+00	3.23E−01	1.32E+02	2.88E−17
ER(−)	24	MR Egger	9.72E+00	1.67E+04	1.25E−01	2.22E+09	1.21E−01	3.25E+01	6.90E−02
		Weighted median	− 4.86E+00	7.73E−03	1.87E−05	3.20E+00	1.14E−01		
		IVW	− 2.73E+00	6.53E−02	4.86E−04	8.78E+00	2.75E−01	3.99E+01	1.56E−02
*Lung cancers*									
Lung	25	MR Egger	− 5.42E−03	9.95E−01	9.77E−01	1.01E+00	5.52E−01	2.07E+01	5.97E−01
		Weighted median	− 7.87E−03	9.92E−01	9.80E−01	1.00E+00	1.92E−01		
		IVW	− 6.19E−03	9.94E−01	9.86E−01	1.00E+00	1.06E−01	2.08E+01	6.53E−01
NSCLC	24	MR Egger	− 2.83E+00	5.88E−02	2.12E−07	1.63E+04	6.62E−01	2.76E+01	0.1910275
		Weighted median	− 7.83E−01	4.57E−01	3.03E−04	6.89E+02	8.34E−01		
		IVW	1.39E−01	1.15E+00	6.62E−03	1.99E+02	9.58E−01	2.79E+01	2.20E−01
SCLC	24	MR Egger	− 7.91E+00	3.66E−04	1.26E−18	1.06E+11	6.46E−01	1.93E+01	6.29E−01
		Weighted median	− 1.29E+01	2.53E−06	1.97E−15	3.24E+03	2.28E−01		
		IVW	− 4.91E+00	7.35E−03	6.55E−09	8.25E+03	4.89E−01	1.93E+01	6.84E−01
*Skin cancers*									
Skin	16	MR Egger	− 1.06E−02	9.89E−01	9.47E−01	1.03E+00	6.47E−01	1.72E+01	2.47E−01
		Weighted median	− 4.75E−03	9.95E−01	9.71E−01	1.02E+00	7.05E−01		
		IVW	3.59E−03	1.00E+00	9.84E−01	1.02E+00	7.10E−01	1.78E+01	2.75E−01
Melanoma	24	MR Egger	4.95E+00	1.42E+02	1.05E−18	1.92E+22	8.36E−01	2.36E+01	3.70E−01
		Weighted median	2.38E+01	2.24E+10	1.90E−02	2.65E+22	9.29E−02		
		IVW	5.54E+00	2.56E+02	1.44E−06	4.55E+10	5.67E−01	2.36E+01	4.28E−01
Non-melanoma	24	MR Egger	− 2.39E+00	9.21E−02	3.71E−05	2.29E+02	5.56E−01	5.65E+01	7.30E−05
		Weighted median	− 4.90E−01	6.12E−01	3.01E−02	1.25E+01	7.50E−01		
		IVW	− 6.49E−01	5.22E−01	2.10E−02	1.30E+01	6.92E−01	5.71E+01	1.00E−04
*Other cancers*									
Kidney	24	MR Egger	2.75E+00	1.57E+01	8.17E−06	3.01E+07	7.13E−01	1.90E+01	6.45E−01
		Weighted median	1.48E+00	4.40E+00	3.98E−04	4.87E+04	7.55E−01		
		IVW	3.07E+00	2.15E+01	5.05E−02	9.11E+03	3.21E−01	1.90E+01	7.00E−01
Bladder	25	MR Egger	1.26E−02	1.01E+00	9.63E−01	1.06E+00	6.29E−01	1.63E+01	8.43E−01
		Weighted median	2.67E−02	1.03E+00	9.93E−01	1.06E+00	1.24E−01		
		IVW	1.88E−02	1.02E+00	9.97E−01	1.04E+00	8.65E−02	1.64E+01	8.75E−01
Bone/cartilage	24	MR Egger	− 1.34E+00	2.61E−01	7.12E−21	9.56E+18	9.54E−01	2.70E+01	2.11E−01
		Weighted median	− 1.95E+00	1.42E−01	3.60E−12	5.58E+09	8.75E−01		
		IVW	− 1.22E+01	4.84E−06	4.13E−14	5.68E+02	1.97E−01	2.73E+01	2.41E−01
Thyroid gland	24	MR Egger	− 6.72E+00	1.20E−03	6.00E−10	2.40E+03	3.74E−01	1.89E+01	6.54E−01
		Weighted median	4.42E+00	8.33E+01	7.96E−03	8.71E+05	3.49E−01		
		IVW	1.78E+00	5.93E+00	1.40E−02	2.52E+03	5.64E−01	2.05E+01	6.14E−01
Brain	24	MR Egger	6.43E+00	6.20E+02	5.36E−07	7.18E+11	5.52E−01	1.66E+01	7.87E−01
		Weighted median	1.63E+00	5.10E+00	2.02E−05	1.29E+06	7.97E−01		
		IVW	2.64E−01	1.30E+00	2.13E−04	7.97E+03	9.53E−01	1.70E+01	8.11E−01

*P value < 0.05; IVW, inverse variance weighted method; OR, odds ratio; OR_lo95%, the lower margin of OR’s 95% confidence interval; OR_up95%, the upper margin of OR’s 95% confidence interval; IVs, instrumental variants; *P*_h_-value, the *P* value of heterogeneity, heterogeneity existed when *P*_h_-value < 0.05

#### Median analysis results

The following 20 probable mediators were investigated to figure out whether MR is shown to be causally related to both the effect of metformin use on them (step one) and the mediators' effects on HER-positive breast cancer (step two): inflammation-related factors (white blood cell counts and C-reactive protein), body shape-related index (BMI, weight, waist circumference, body fat percentage, visceral adipose tissue volume, and abdominal subcutaneous adipose tissue volume), metabolism-related biomarkers (HbA1c, fasting insulin, and fasting glucose) and sex hormone-related biomarkers (SHBG, estradiol, total testosterone levels, and bioavailable testosterone levels). As shown in Table 2, we determined that metformin treatment had a causal influence on HDL cholesterol, LDL cholesterol, SHBG, total testosterone, bioavailable testosterone, estradiol, and fasting glucose levels. MR analyses were further conducted to evaluate the causal effect of the seven mediators above on HER-positive breast cancer (Table 3). Significant causal associations was exhibited in total testosterone levels (Beta: 0.4058, 95% CI: 0.0562 to 0.7556, *P *value: 0.0229). Hence, total testosterone (TT) levels was selected for mediation effect calculation (Additional file 1: Figs. S3–S10).

**Table 2:** Genetic causal effect of metformin use on potential mediators

Potential mediators	GWAS identifier/reference	Beta1	Lo_95CI	Up_95CI	*P*1_value
BMI	ebi-a-GCST006802 [45]	− 4.00E−01	− 1.51E+00	7.10E−01	4.80E−01
Weight	ukb-b-11842	− 1.72E−01	− 2.02E+00	1.68E+00	8.55E−01
Waist circumference	ieu-a-67 [46]	5.12E−01	− 1.70E+00	2.73E+00	6.50E−01
ASATV	ebi-a-GCST90016672 [47]	− 2.40E−01	− 2.56E+00	2.08E+00	8.40E−01
VATV	ebi-a-GCST90016671 [47]	2.41E−01	− 1.61E+00	2.09E+00	7.99E−01
Whole body fat-free mass	ukb-b-13354	− 6.68E−02	− 1.32E+00	1.19E+00	9.17E−01
Body fat percentage	ukb-b-8909	− 1.82E−01	− 1.45E+00	1.08E+00	7.78E−01
WBC	ebi-a-GCST004610 [48]	− 1.21E−01	− 1.05E+00	8.11E−01	7.99E−01
CRP	ieu-b-4764	− 1.99E−01	− 1.35E+00	9.52E−01	7.35E−01
HDL cholesterol	ieu-b-109 [49]	− 2.38E+00	− 4.40E+00	− 3.62E−01	2.08E−02*
LDL cholesterol	ieu-b-5089	− 2.37E+00	− 3.21E+00	− 1.54E+00	2.38E−08*
Total cholesterol	ieu-a-301 [50]	− 6.69E−01	− 3.02E+00	1.68E+00	5.77E−01
SHBG	ebi-a-GCST90012111 [51]	− 1.50E+00	− 2.08E+00	− 9.24E−01	3.57E−07*
Total testosterone	ebi-a-GCST90012114 [51]	− 6.04E−01	− 9.99E−01	− 2.08E−01	2.76E−03*
Bioavailable testosterone	ebi-a-GCST90012102 [51]	9.28E−01	3.35E−01	1.52E+00	2.15E−03*
Estradiol levels	ebi-a-GCST90012105 [51]	− 1.23E−01	− 2.32E−01	− 1.31E−02	2.82E−02*
Fasting insulin	ebi-a-GCST90002238 [52]	− 3.85E−01	− 1.34E+00	5.68E−01	4.28E−01
Fasting glucose	ebi-a-GCST005186 [53]	2.65E+00	1.06E+00	4.23E+00	1.06E−03*
HbA1c	ieu-b-104 [54]	− 3.96E−01	− 9.88E−01	1.97E−01	1.90E−01
Telomere length	ieu-b-4879	− 6.25E−03	− 4.43E−01	4.30E−01	9.78E−01

All the results above were derived from the IVW method.

*P1_value < 0.05; Lo_95CI, the lower margin of beta 1’s 95% confidence interval; Up_95CI, the upper margin of beta 1’s 95% confidence interval; SHBG,Sex hormone-binding globulin; VATV, Visceral adipose tissue volume; ASATV, Abdominal subcutaneous adipose tissue volume; HDL, high-density lipoprotein; LDL, low-density lipoprotein; WBC, White blood cell

**Table 3:** Genetic causal effect of potential mediators on HER-positive breast cancer

Potential mediators	Beta2	Lo_95CI	Up_95CI	P2_value
HDL cholesterol	2.85E−02	− 8.63E−02	1.43E−01	6.27E−01
LDL cholesterol	6.97E−02	− 5.83E−02	1.98E−01	2.86E−01
SHBG	− 8.30E−02	− 3.50E−01	1.84E−01	5.42E−01
Total testosterone	4.06E−01	5.62E−02	7.56E−01	2.29E−02*
Bioavailable testosterone	1.73E−01	3.61E−02	3.81E−01	1.05E−01
Estradiol levels	1.34E+00	− 6.24E−01	3.30E+00	1.82E−01
Fasting glucose	− 1.66E−01	− 4.72E−01	1.39E−01	2.86E−01

All the results above were derived from the IVW method.

*P2_value < 0.05; Lo_95CI, the lower margin of beta 2’s 95% confidence interval; Up_95CI, the upper margin of beta 2’s 95% confidence interval; SHBG, Sex hormone-binding globulin; HDL, high-density lipoprotein; LDL, low-density lipoprotein

In the MVMR of metformin-TT-HER(+) breast cancer, the direct effect of metformin on HER(+) breast cancer was OR 0.0992 (95% CI: 0.0038 to 2.5986, *P *value: 0.1655) after being adjusted by TT levels, and the direct effect of TT on HER(+) breast cancer was OR 1.5964 (95% CI: 1.1334 to 2.2486, *P *value: 0.0074) after being adjusted by metformin use (Additional file 1: Table S2). The mediation effect of TT levels was 24.52%.

### Review of previous clinical studies

With the help of the three databases mentioned above, we listed the literature reviews of clinical studies concerning metformin use on breast cancer in Table 4, both therapeutic and preventive effect were reviewed here.

**Table 4:** Literature review of clinical studies concerning metformin use on breast cancer

Study design	Metformin dose	T2DM	Population	Sample size	Age (years)	Outcomes	References
*Clinical studies of the treatment effect of metformin on breast cancer*							
RCT, Phase III	850 mg b.i.d for 5 years	No	American Indian or Alaska Native (0.5%)	ER/PgR−:	Median (range)	ER/PgR−: not significant	Goodwin et al. [55]
				Case: 1268/control: 1265	Case: 51 (25–74)	DFS:HR = 1.01 (95% CI 0.84–1.21)	
			Asian (2.8%)			OS:HR = 1.10 (95% CI 0.86–1.41)	
					Control: 52 (23–74)		
			Black or African American (4.3%)				
			Hispanic (4.9%)	ER/PgR+:	Case: 52 (25–74)	ER/PgR+: not significant	
				Case: 556/control: 560		DFS:HR = 1.01 (95% CI 0.79–1.30)	
			Native Hawaiian or Pacific Islander (0.4%)			OS:HR = 0.89 (95% CI 0.64–1.23)	
					Control: 53 (25–74)		
			White non-Hispanic (85.7%)				
RCT, Phase II	1000 mg b.i.d for 1.5 years	No	Egyptian	Case: 36/control: 38	Mean (± SD)	Not significant	Barakat et al. [5]
					Case: 49.14 ± 11.22	PCR:OR = 2.429 (95% CI 0.662–8.914)	
					Control: 47.13 ± 10.53	ORR:OR = 1.912 (95% CI 0.655 –5.585)	
						CCR:OR = 3.269 (95% CI 0.921–11.606)	
RCT, Phase II	850 mg b.i.d for 6 weeks	Partial	Chinese	Case: 41/control: 35	Case: ≤ 50 (42.0%), > 50 (58.0%)	Not significant	Huang et al. [56]
						tpCR (*P* = 0.777)	
					Control: ≤ 50 (40.5%), > 50 (59.5%)	bpCR (*P* = 0.956)	
						Clinical response (*P* = 0.930)	
RCT, Phase II	850 mg b.i.d for 151 days	No	Canadian	Case: 22/control: 17	Median (range)	Not significant	Pimentel et al. [57]
					Case: 55 (39–75)	PFS:HR = 1.2 (95% CI 0.63–2.31)	
					Control: 57 (41–73)	OS:HR = 1.68 (95% CI 0.79–3.55)	
RCT, Phase II	1000 mg b.i.d for 39.6 months	No	Italian	HER2−:	Case: ≤ 50 (26.0%), > 50 (74.0%)	Not significant	Nanni et al. [58]
				Case: 57/control: 65		PFS:HR = 1.09 (95% CI 0.75–1.58)	
					Control: ≤ 50 (21.0%), > 50 (79.0%)	OS:HR = 0.81 (95% CI 0.50–1.30)	
						ORR (*P* = 0.901)	
RCT, Phase II	1000 mg b.i.d for 6.3 months	No	Black or African American (9%)	HR+, HER2−:	Median (range)	Median PFS: 6.3 months (95% CI 3.8–11.3 months)	Yam et al. [59]
			White non-Hispanic (91%)	22	57.2 (37.6–70.5)	Median OS: 28.8 months (95% CI 17.5–59.7 months)	
RCT, Phase II	500 mg b.i.d until endpoint	No	Egyptian	Case: 57/control: 50	Mean (± SD)	Not significant	Essa et al. [60]
					Case: 49.56 ± 12.53	RR: *P* = 0.205	
					Control: 48.40 ± 12.61	PFS: *P* = 0.753	
Case–control observational study	Not mentioned	Yes	Belgium	HR+:	case: ≤ 50 (13.08%), > 50 (86.92%)	Signficant in HR + group	Sonnenblick et al. [61]
				Case: 136/control: 95		DFS:HR+:HR = 0.46 (95% CI 0.24–0.89);	
						HR−:HR = 0.99 (95% CI 0.53–1.87)	
				HR−:	Control: ≤ 50 (18.82%), > 50 (81.18%)	DFS:HR+:HR = 0.29 (95% CI 0.14–0.63);	
				Case: 124/control: 91		HR−:HR = 1.05 (95% CI 0.51–2.14)	
						OS:HR+:HR = 0.27 (95% CI 0.10–0.71);	
						HR−:HR = 1.09 (95% CI 0.48–2.47)	
Cohort study	Not mentioned	Partial	Australian	6717	Mean (± SD)	Significant	Feng et al. [62]
					66.8 (± 9.8)	Each 1-year adherence to metformin reduces cancer-specific mortality: adjusted HR = 0.95 (95% CI 0.93–0.97)	
Retrospective study	Not mentioned	Yes	Korean	Case: 11,490/control: 22,265	Case: ≤ 65 (70.6%), > 65 (29.4%)	OS:HR = 0.83 (95% CI 0.73–0.94);	Kim et al. [63]
					Control: ≤ 65 (68.8%), > 65 (31.2%)		
Retrospective study	Not mentioned	Partial	Chinese	Case: (diabetic) 312/control (non-diabetic): 3139		Significant	Hui et al. [64]
					Case: ≤ 55 (41.7%), > 55 (58.3%)	OS:HR = 0.386 (95% CI 0.248–0.601)	
					Control: ≤ 55 (67.3%), > 55 (32.7%)	DFS:HR = 0.384 (95% CI 0.247–0.598)	
Retrospective study	Not mentioned	Yes	Finnish	3165	Median (range)	Not significant	Hosio et al. [65]
					72 (64–79)	HR= 0.92 (95% CI 0.64–1.31)	
Retrospective study	Not mentioned	Partial	Egyptian	Case: 25/control (diabetic): 14/control (non-diabetic): 400	Median (range)	Significant	El-Benhawy et al. [66]
					Case: 55 (36–75)	DFS: compared with diabetic control group: *P* = 0.0001; compared with non-diabetic control group: *P* = 0.0249	
					Control (diabetic): 53 (43–64)	OS: compared with diabetic control group: *P* = 0.0032; compared with non-diabetic control group: *P* = 0.0350	
					Control (non-diabetic): 53 (33–65)		
Retrospective study	Not mentioned	Yes	Korean	Case: 202/control: 184	Median (range)	Significant	Kim et al. [67]
					Case: 55 (± 10.5)	Higher metastasis risk in diabetic control group: HR= 5.37 (95% CI 1.88–15.28)	
					Control: 59 (± 10.2)	Higher breast cancer death in control group: HR= 6.51 (95% CI 1.88 to 15.28)	
*Observational studies of the prevention effect of metformin on breast cancer*							
Prospective study	Not mentioned	Yes	Non-Hispanic white: case: (69%); control: (86%)	Case: (diabetic)111/control (non-diabetic): 1800	Mean (± SD)	Not Significant in overall breast cancer risk (HR= 0.98; 95% CI 0.83–1.15)	
			Non-Hispanic black: case: (18%); control: (8%)		Case: 58.2 ± 8.2	Significant in ER +	Park et al. [68]
			Other: case: (13%); control: (7%)		Control: 55.1 ± 8.9	HR= 0.86; (95% CI 0.70–1.05)	
						≥ 10 years metformin use: HR 0.62 (95% CI 0.38–1.01)	
Case–control observational study	Not mentioned	Yes	White: case: (80.58%); control: (65.60%)	Case: 690/control: 2747	Mean (± SD)	Significant	
			Black: case: (7.54%); control: (10.74%)		Case: 70.06 ± 5.00	> 500 mg/day metformin use had 39% lower odds of HR + /HER2- breast cancer (OR = 0.61, 95% CI 0.46–0.82)	Chikermane et al. [69]
			Asian: case: (5.22%); control: (11.47%)		Control: 69.92 ± 4.84		
			Hispanic: case: (2.17%); control: (7.10%)				
			Other: case: (4.49%); control: (5.10%)				

RCT, randomized clinical trial; T2DM, type 2 diabetes; SD, standard deviation; OR, odds ratio; CI, confidential interval; PFS, progression-free survival; DFS, disease-free survival; ORR, objective response rate; OS, overall survival; HR, hormone receptor/hazard ratio; tpCR, total pathological complete response; bpCR, pathological complete response in the breast; ER, estrogen receptor; PgR, progesterone receptor

## Discussion

In addition to its well-established role in reducing persistently high plasma glucose and insulin levels, metformin stands out as a promising candidate for the prevention and treatment of malignant tumors. Recent years have witnessed the promising efficacy of metformin in the management of several types of cancer. However, clinical outcomes have been inconsistent [5, 20, 21]. To optimize the anti-tumor effect of metformin, researchers have focused on the underlying mechanisms for decades. As previously mentioned, numerous pathways and mediating molecules connecting metformin to its effects on cancer have come to light. These include the activation of AMPK-related pathways [22, 23], the promotion of apoptotic cell death in cancer cells [24, 25], and the inhibition of mitochondrial metabolism [26, 27]. These mechanistic studies have shed light on the pivotal role of metformin in cancer therapy and the regulatory pathways involved. Their findings hold significant promise for advancing future clinical management and pharmaceutical development in the field of cancer treatment.

There is a growing focus on investigating the genetic aspects of metformin's role in cancer treatment. In this study, we employed MR analysis to uncover the genetically predicted connections between metformin usage and the risk of common cancers. Unlike traditional clinical studies, MR analysis offers several advantages. It helps eliminate the influence of irrelevant confounding factors and environmental exposures, mitigates the impact of reverse causality, and enhances the strength of evidence for causal inference [28]. As a result, MR analysis stands as a relatively reliable and cost-effective method, leveraging global genome databases to advance our understanding of the relationship between metformin and cancer risk.

Several MR studies have demonstrated the genetic influence of metformin on a variety of diseases. For instance, Zhou et al.'s MR analysis examined the relationship between metformin use and lung cancer risk, finding no genetic causality between the two, a result consistent with our own findings [29]. Modest genetic associations were also reported in the context of breast cancer and prostate cancer [30]. Notably, the MR study on breast cancer encompassed overall, estrogen receptor (ER)-positive, and ER-negative subtypes, yielding results congruent with our research. Beyond cancers, the causal role of metformin on other diseases has been assessed as well. Zhang et al. reported the protective causal relationship between metformin targets and osteoarthritis, highlighting AMPK and GDF-15 as promising targets for osteoarthritis treatment [31]. However, GDF-15 as a therapeutic target of metformin might increase the risk of gallstone disorders [32]. Given metformin's multiple drug targets, which cannot be simplified into one or two specific targets, the accuracy of drug target MR analysis for explaining its therapeutic effects may be limited.

The relationship between sex hormone levels and breast cancer is indeed intricate and has been the focus of extensive research. A comprehensive review study, encompassing 44 breast cancer research studies, revealed that the risk of breast cancer increased with the use of oral contraceptives [33]. Furthermore, this risk was positively correlated with the duration of oral contraceptive use, shedding light on the association between estrogen and progestogen and the prevalence of breast cancer [34]. Testosterone, another sex hormone, also plays a significant role in the development of breast cancer. Evidence from a case–control analysis within the European Prospective Investigation into Cancer and Nutrition cohort established a link between elevated blood testosterone concentrations and an increased incidence of breast cancer (OR: 1.73, 95% CI: 1.16 to 2.57; *P *value: 0.01) among premenopausal individuals [35]. Similar findings have been reported by researchers from various countries [36–38]. Moreover, two-sample MR studies conducted by UK scientists underscored the potential impact of sex steroid hormones on breast cancer risk, These studies pointed out that testosterone and bioavailable testosterone could elevate the risk of both overall and estrogen receptor-positive (ER-positive) breast cancer [39, 40]. These findings align closely with our own research, solidifying the notion of a robust association between testosterone levels and breast cancer risk.

Remarkably, our current study unveiled a novel finding, demonstrating that metformin has the potential to reduce the risk of HER-positive (HER+) breast cancer, and this reduction is partially mediated through its impact on total testosterone levels. The testosterone reduction effect of metformin has been observed in previous reports [41, 42]. Some clinical trials have administrated metformin on non-diabetic breast cancer women, ending with a significant reduction of both insulin and testosterone levels [43, 44]. Furthermore, metformin primarily lowered estradiol levels by diminishing testosterone levels, and these hormonal alterations may hold relevance in certain clinical contexts. This underscores the multifaceted effects of metformin on hormonal regulation and its potential implications in breast cancer prevention and treatment.

Our study boasts several notable strengths. To our knowledge, this is the first study figuring out the genetic effect of metformin use on multiple prevalent cancer risks. And the mediators on the genetic pathway were clarified, and their mediating effects were calculated. Moreover, the genetic information incorporated in this study is giant, which increases the credibility of the conclusion.

While our study presents valuable insights, it's important to acknowledge its limitations. First, to ascertain the consistency of genetic background, this MR analysis only concluded European populations, which could not be extended to other ethnicities. Second, MR analysis of tumors with a small number of instances was less accurate (fewer than 1000). For the validation analysis, more genetic data from large samples need to be added. The association between metformin use and other malignancies cannot be determined at this time; however, this will be clarified in follow-up research.

## Conclusion

The current MR study revealed that metformin use could genetically shield individuals from HER-positive breast cancer, which was mediated by total testosterone levels. Further investigation is required to determine whether metformin-induced changes in total testosterone levels could potentially serve as a predictor or biomarker in HER-positive breast cancer development and progression.

### Supplementary Information

**Additional file 1. Table S1**: SNPs associated with metformin use, which performed as instrumental variants (IVs) in two-sample MR analysis. **Table S2**: The genetic effect obtained from MVMR analysis. TT: total testosterone levels. **Fig. S1**: Scatter plots and funnel plots of metformin use on HER-positive breast cancer. **A** Scatter plots of the genetic association between metformin use and HER-positive breast cancer. The genetic predicted metformin use is associated with a lower risk of HER-positive breast cancer. The slope of each line shows the estimated causal effect of metformin use on HER-positive breast cancer for each approach. **B** Funnel plots showing the statistical association between metformin use and the risk of HER-positive breast cancer. **Fig. S2**: Leave-one-out analysis and Forest plots results. **A** Leave-one-out analysis of sensitivity test. After one by one eliminating the IVs, calculate the MR outcomes for the remaining IVs. **B** Forest plot of the causal effects of metformin use associated SNPs on HER-positive breast cancer. **B** Showed the Mendelian randomization estimated effects sizes for metformin use on HER-positive breast cancer. **Fig. S3**: Leave-one-out analysis result of metformin use on total testosterone levels. **Fig. S4**: Leave-one-out analysis result of total testosterone levels on HER-positive breast cancer. **Fig. S5**: Scatter plot of metformin use on total testosterone levels. **Fig. S6**: Scatter plot of total testosterone levels on HER-positive breast cancer. **Fig. S7**: Forest plot of metformin use on total testosterone levels. **Fig. S8**: Forest plot of total testosterone levels on HER-positive breast cancer. **Fig. S9**: Funnel plot of metformin use on total testosterone levels. **Fig. S10**: Funnel plot of total testosterone levels on HER-positive breast cancer.

## Data Availability

All of the genetic data used in this work was publicly available. The relevant data can be found here: Open GWAS summary dataset (https://gwas.mrcieu.ac.uk/); UK Biobank (https://www.ukbiobank.ac.uk/); FinnGen database (https://www.finngen.fi/).
