# Supplementary material for: Genetic effect of metformin use on risk of cancers: evidence from Mendelian randomization analysis
Source: Diabetol Metab Syndr. 2023 Dec 6;15:252. doi: 10.1186/s13098-023-01218-3 (PMC10699048; doi:10.1186/s13098-023-01218-3)
Supplement: Supplementary file 1 — Additional file 1. Table S1: SNPs associated with metformin use, which performed as instrumental variants (IVs) in two-sample MR analysis. Table S2: The genetic effect obtained from MVMR analysis. TT: total testosterone levels. Fig. S1: Scatter plots and funnel plots of metformin use on HER-positive breast cancer. A Scatter plots of the genetic association between metformin use and HER-positive breast cancer. The genetic predicted metformin use is associated with a lower risk of HER-positive breast cancer. The slope of each line shows the estimated causal effect of metformin use on HER-positive breast cancer for each approach. B Funnel plots showing the statistical association between metformin use and the risk of HER-positive breast cancer. Fig. S2: Leave-one-out analysis and Forest plots results. A Leave-one-out analysis of sensitivity test. After one by one eliminating the IVs, calculate the MR outcomes for the remaining IVs. B Forest plot of the causal effects of metformin use associated SNPs on HER-positive breast cancer. B Showed the Mendelian randomization estimated effects sizes for metformin use on HER-positive breast cancer. Fig. S3: Leave-one-out analysis result of metformin use on total testosterone levels. Fig. S4: Leave-one-out analysis result of total testosterone levels on HER-positive breast cancer. Fig. S5: Scatter plot of metformin use on total testosterone levels. Fig. S6: Scatter plot of total testosterone levels on HER-positive breast cancer. Fig. S7: Forest plot of metformin use on total testosterone levels. Fig. S8: Forest plot of total testosterone levels on HER-positive breast cancer. Fig. S9: Funnel plot of metformin use on total testosterone levels. Fig. S10: Funnel plot of total testosterone levels on HER-positive breast cancer. [file 13098_2023_1218_MOESM1_ESM.docx]

| **SNP** | **Chr** | **Sample size** | **Effect allele** | **Other allele** | **EAF** | **Beta** | **SE** | **P_value** | **F_statistic** |
| --- | --- | --- | --- | --- | --- | --- | --- | --- | --- |
| rs10196106 | 2 | 337159 | T | G | 1.01E-01 | -4.50E-03 | 7.14E-04 | 2.98E-10 | 3.97E+01 |
| rs2972143 | 2 | 337159 | G | A | 6.47E-01 | 2.33E-03 | 3.97E-04 | 4.51E-09 | 3.44E+01 |
| rs1496653 | 3 | 337159 | G | A | 2.04E-01 | -3.01E-03 | 4.70E-04 | 1.42E-10 | 4.11E+01 |
| rs9808924 | 3 | 337159 | A | G | 3.14E-01 | 3.79E-03 | 4.08E-04 | 1.62E-20 | 8.62E+01 |
| rs10001190 | 4 | 337159 | G | A | 6.30E-01 | 2.82E-03 | 3.93E-04 | 6.93E-13 | 5.16E+01 |
| rs115576427 | 5 | 337159 | G | A | 4.98E-02 | 5.53E-03 | 8.76E-04 | 2.77E-10 | 3.98E+01 |
| rs459193 | 5 | 337159 | G | A | 7.47E-01 | 2.51E-03 | 4.35E-04 | 7.46E-09 | 3.34E+01 |
| rs7766070 | 6 | 337159 | A | C | 2.64E-01 | 3.29E-03 | 4.30E-04 | 1.79E-14 | 5.88E+01 |
| rs849142 | 7 | 337159 | C | T | 5.06E-01 | -2.56E-03 | 3.79E-04 | 1.50E-11 | 4.55E+01 |
| rs13266634 | 8 | 337159 | T | C | 3.11E-01 | -2.61E-03 | 4.10E-04 | 1.96E-10 | 4.05E+01 |
| rs7018475 | 9 | 337159 | G | T | 2.56E-01 | 2.85E-03 | 4.34E-04 | 5.19E-11 | 4.31E+01 |
| rs10965246 | 9 | 337159 | C | T | 1.77E-01 | -4.60E-03 | 4.97E-04 | 2.15E-20 | 8.57E+01 |
| rs810517 | 10 | 337159 | T | C | 4.62E-01 | -2.60E-03 | 3.80E-04 | 7.14E-12 | 4.70E+01 |
| rs34872471 | 10 | 337159 | C | T | 2.91E-01 | 8.94E-03 | 4.17E-04 | 8.17E-102 | 4.59E+02 |
| rs34744311 | 10 | 337159 | T | C | 3.77E-01 | -2.71E-03 | 3.92E-04 | 4.45E-12 | 4.79E+01 |
| rs11257655 | 10 | 337159 | T | C | 2.09E-01 | 2.66E-03 | 4.67E-04 | 1.15E-08 | 3.26E+01 |
| rs2237895 | 11 | 337159 | C | A | 4.16E-01 | 2.57E-03 | 3.84E-04 | 2.00E-11 | 4.50E+01 |
| rs11602873 | 11 | 337159 | T | A | 1.58E-01 | -3.02E-03 | 5.19E-04 | 6.06E-09 | 3.38E+01 |
| rs76895963 | 12 | 337159 | G | T | 1.93E-02 | -1.22E-02 | 1.47E-03 | 1.30E-16 | 6.85E+01 |
| rs649698 | 13 | 337159 | C | G | 4.34E-01 | 2.99E-03 | 3.83E-04 | 5.22E-15 | 6.12E+01 |
| rs12889801 | 14 | 337159 | T | C | 4.83E-01 | -2.12E-03 | 3.79E-04 | 2.50E-08 | 3.11E+01 |
| rs4932264 | 15 | 337159 | C | T | 7.30E-01 | -2.39E-03 | 4.27E-04 | 2.24E-08 | 3.13E+01 |
| rs1421085 | 16 | 337159 | C | T | 4.02E-01 | 3.35E-03 | 3.87E-04 | 4.67E-18 | 7.50E+01 |
| rs11658063 | 17 | 337159 | G | C | 6.02E-01 | -2.88E-03 | 3.91E-04 | 1.59E-13 | 5.45E+01 |
| rs9957264 | 18 | 337159 | A | C | 1.65E-01 | -2.93E-03 | 5.12E-04 | 1.05E-08 | 3.27E+01 |
| rs1800961 | 20 | 337159 | T | C | 3.15E-02 | 6.02E-03 | 1.08E-03 | 2.85E-08 | 3.08E+01 |

**Supplementary Table 1. SNPs associated with metformin use, which performed as instrumental variants (IVs) in two-sample MR analysis.**

|  | **Exposure** | **Outcome** | **nSNP** | **Beta** | **SE** | **OR** | **OR_lo95%** | **OR_up95%** | **P-value** |
| --- | --- | --- | --- | --- | --- | --- | --- | --- | --- |
| Metformin + TT | Metformin | HER(+) Breast cancer | 17 | -2.31E+00 | 1.67E+00 | 9.92E-02 | 3.79E-03 | 2.60E+00 | 1.65E-01 |
| Metformin + TT | TT | HER(+) Breast cancer | 147 | 4.68E-01 | 1.75E-01 | 1.60E+00 | 1.13E+00 | 2.25E+00 | 7.44E-03 |

**Supplementary Table 2. The genetic effect obtained from MVMR analysis.** TT: total testosterone levels.


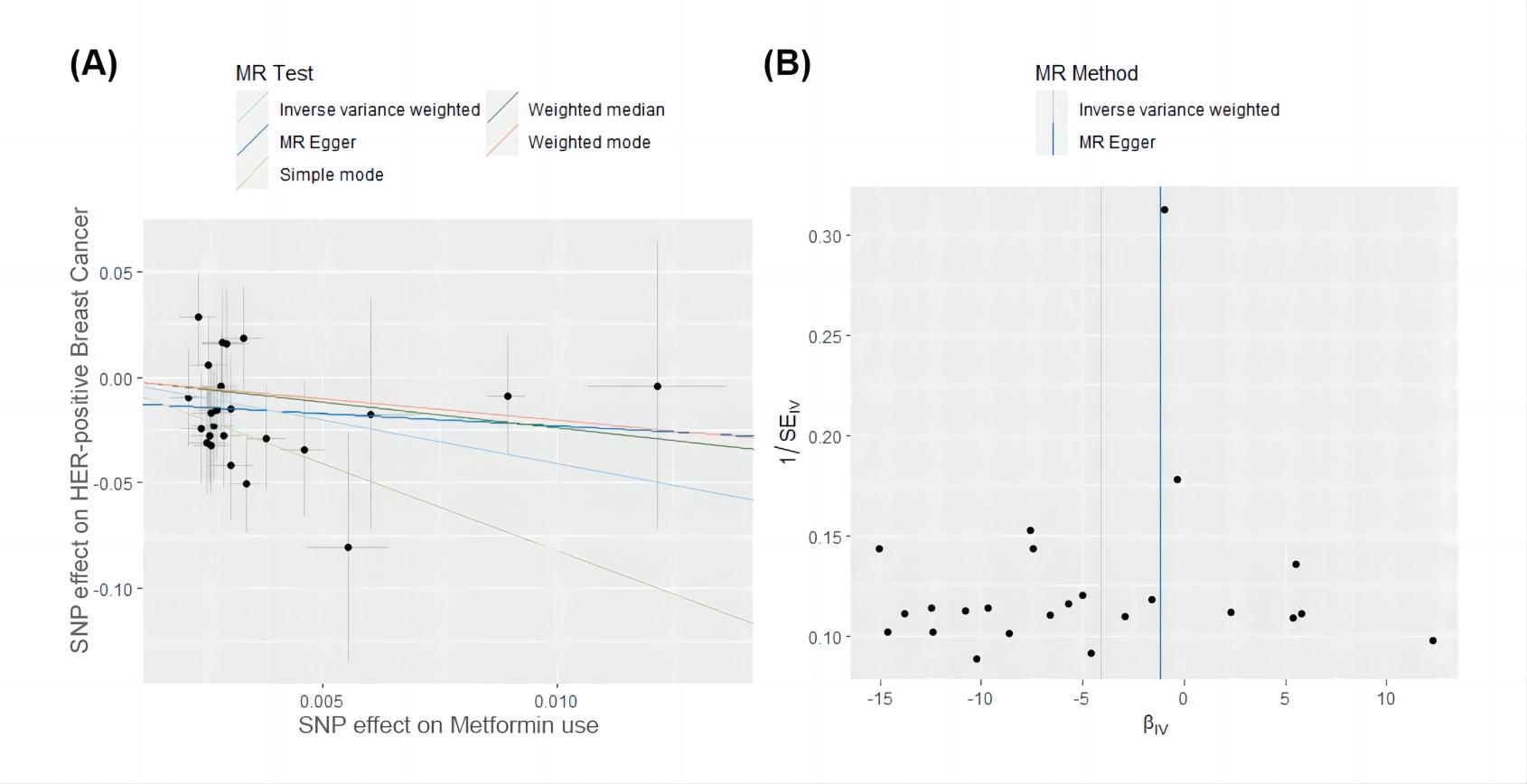


**Supplementary Figure 1. Scatter plots and funnel plots of metformin use on HER-positive breast cancer.** (A) Scatter plots of the genetic association between metformin use and HER-positive breast cancer. The genetic predicted metformin use is associated with a lower risk of HER-positive breast cancer. The slope of each line shows the estimated causal effect of metformin use on HER-positive breast cancer for each approach. (B) Funnel plots showing the statistical association between metformin use and the risk of HER-positive breast cancer.


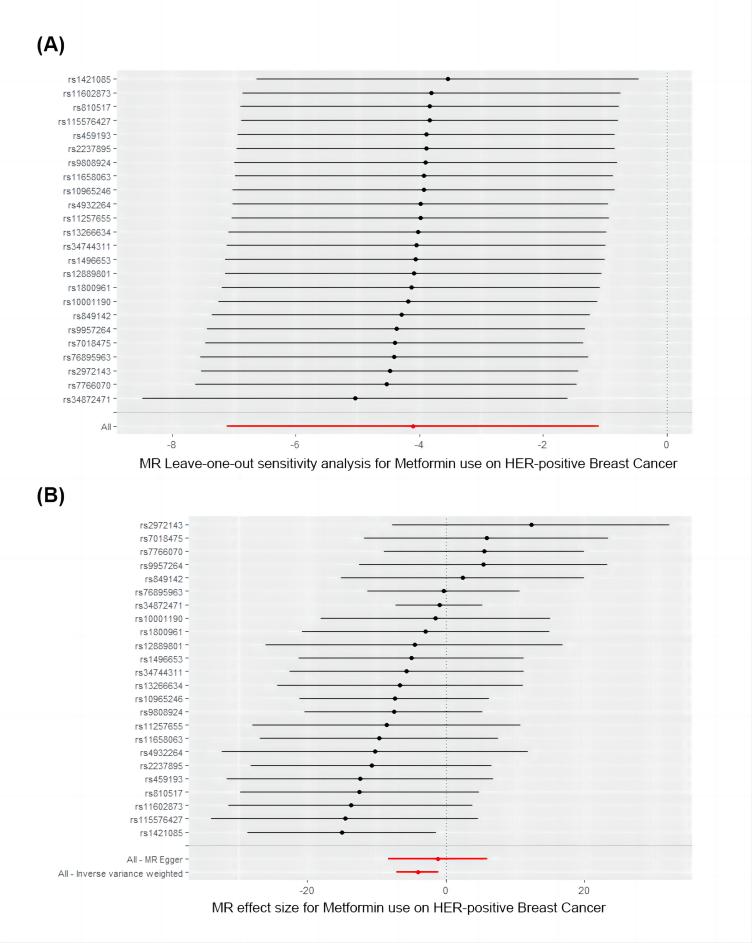


**Supplementary Figure 2. Leave-one-out analysis and Forest plots results.** (A) Leave-one-out analysis of sensitivity test. After one by one eliminating the IVs, calculate the MR outcomes for the remaining IVs. (B) Forest plot of the causal effects of metformin use associated SNPs on HER-positive breast cancer. Figure B showed the Mendelian randomization estimated effects sizes for metformin use on HER-positive breast cancer.


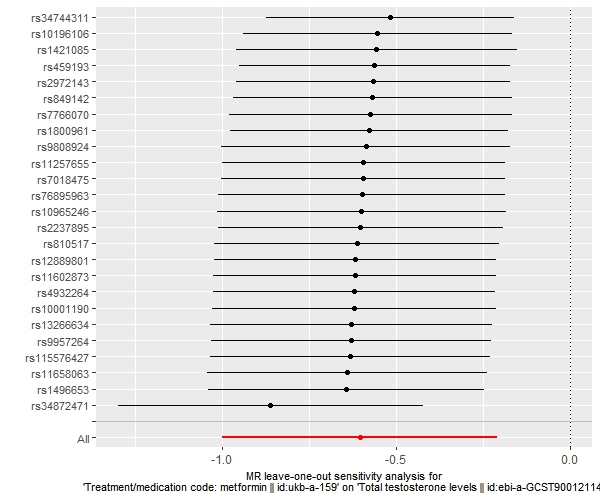


**Supplementary Figure 3. Leave-one-out analysis result of metformin use on total testosterone levels.**

**
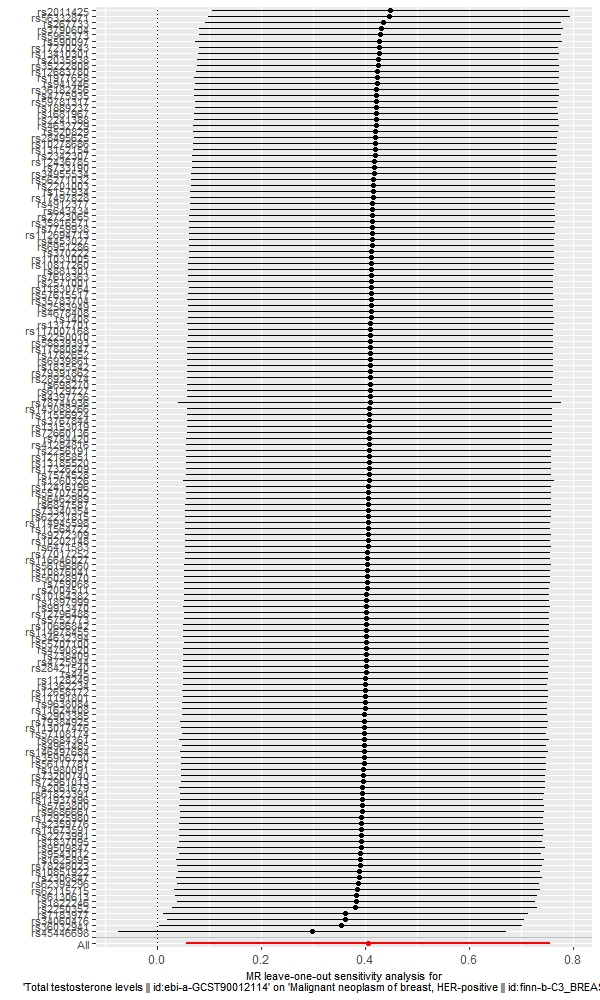
**

**Supplementary Figure 4. Leave-one-out analysis result of total testosterone levels on HER-positive breast cancer.**

**
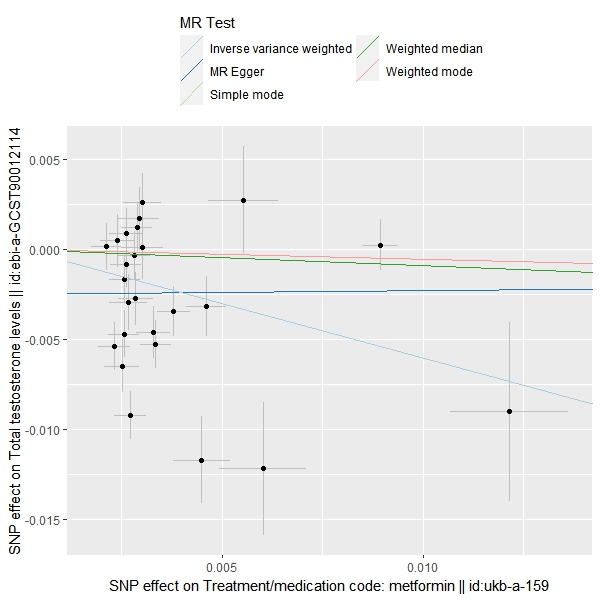
**

**Supplementary Figure 5. Scatter plot of metformin use on total testosterone levels.**

**
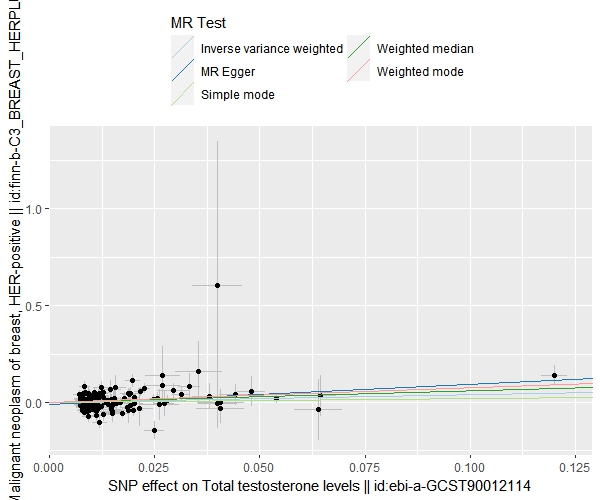
**

**Supplementary Figure 6. Scatter plot of total testosterone levels on HER-positive breast cancer.**

**
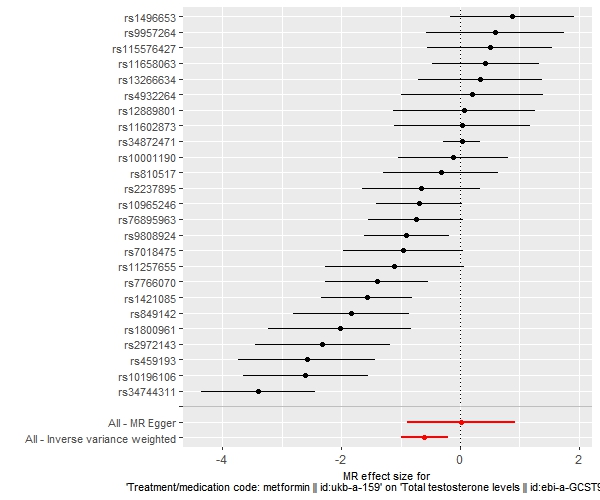
**

**Supplementary Figure 7. Forest plot of metformin use on total testosterone levels.**

**
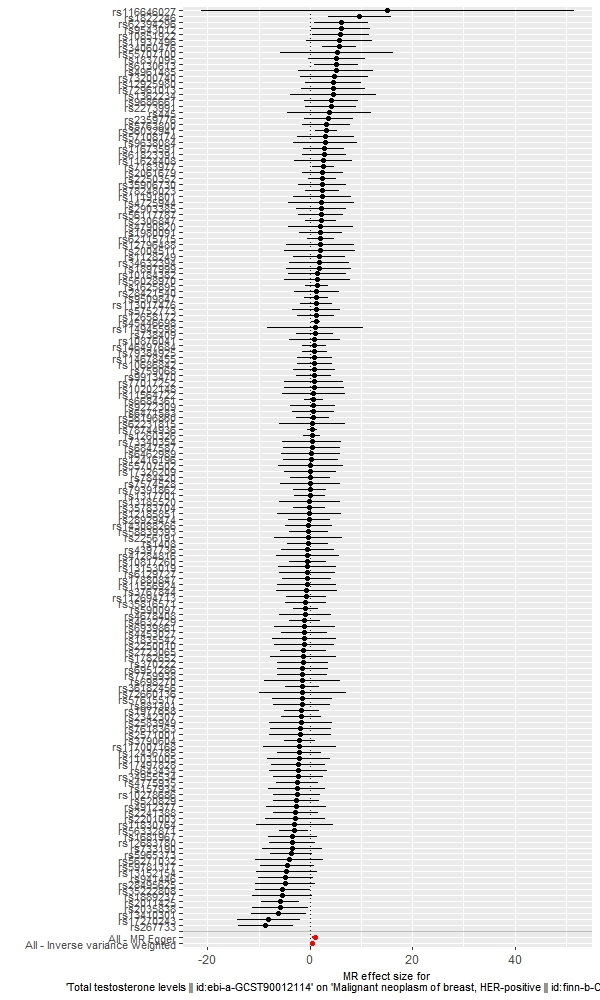
**

**Supplementary Figure 8. Forest plot of total testosterone levels on HER-positive breast cancer.**

**
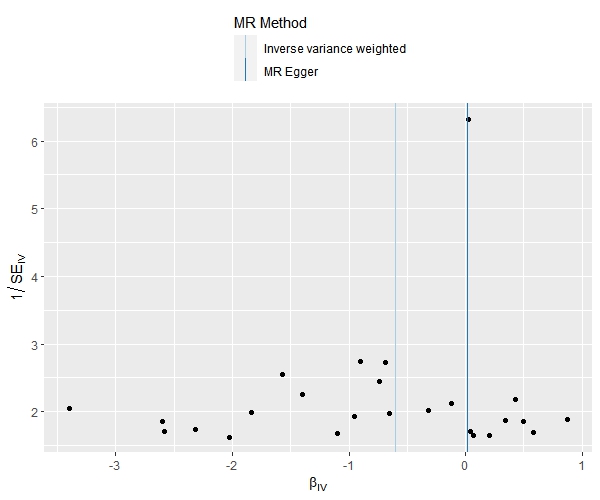
**

**Supplementary Figure 9. Funnel plot of metformin use on total testosterone levels.**

**
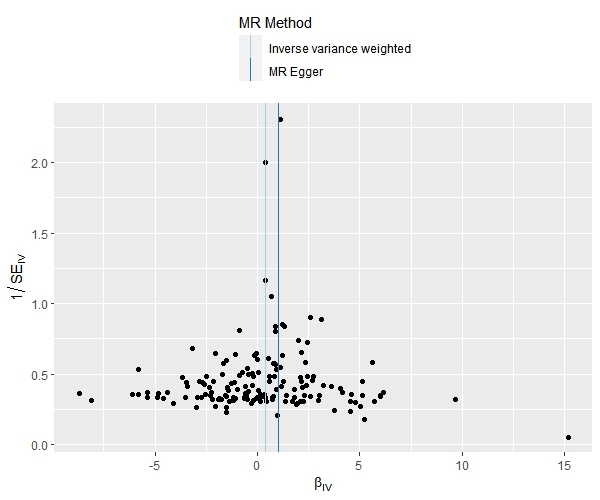
**

**Supplementary Figure 10. Funnel plot of total testosterone levels on HER-positive breast cancer.**
